# Yta7, a chromatin segregase regulated by the cell cycle machinery

**DOI:** 10.1080/23723556.2022.2039577

**Published:** 2022-03-17

**Authors:** Priyanka Bansal, Christoph F. Kurat

**Affiliations:** Molecular Biology Division, Biomedical Center Munich, Ludwig-Maximilians-Universität, Munich, Germany

**Keywords:** Chromosome replication, cell cycle, chromatin biology

## Abstract

We have recently revealed the existence of a cell cycle-regulated chromatin segregase, Yta7 (Yeast Tat-binding Analog 7), involved in chromosome replication. Phosphorylation of Yta7 by S-CDK (S-phase Cyclin-Dependent Kinase) regulates its function. These findings link the cell cycle to chromatin biology and suggest how chromatin-modifying enzymes become S phase-specific.

Genome duplication is one of the most fundamental processes a cell undertakes. Eukaryotic genomes consist of DNA wrapped around histone octamers to form nucleosomes, the basic unit of chromatin.^1^ While vital for many cellular functions, chromatin is a natural barrier for DNA-template processes like transcription, replication, DNA repair or damage. This is because of the inherent stability of nucleosomes. To access DNA packed in chromatin, cells require the help of chromatin-modulating factors like chromatin remodelers or histone chaperones.

In contrast to gene transcription, chromosome replication is special in the way that it takes place exclusively during the S-phase of the cell cycle. It has been shown that ATP-dependent chromatin remodelers such as INO80 complex (INOsitol requiring 80) or ISW1a (Imitation Switch subfamily), the histone chaperone FACT (FAcilitates Chromatin Transcription)/Nhp6 (Non-Histone chromosomal Protein 6) along with the histone acetyl transferases SAGA (Spt-Ada-Gcn5 Acetyltransferase) and NuA4 (NUcleosome Acetyltransferase of H4) are necessary for chromatin replication *in vitro*.^2^ However, *in vivo*, these chromatin factors also play important roles during other processes, most prominently gene transcription. Transcription takes place throughout the cell cycle, and it remains unclear how these chromatin factors were made “S phase-specific” to support chromosome replication.

In our study, we revealed that Yta7 (Yeast Tat-binding Analog 7) is a novel type of chromatin factor, which activity to promote chromosome replication is regulated by phosphorylation by S-CDK.^3^ This makes an important step forward toward understaning how chromatin factors are regulated at specific cell cycle stages. Yta7 was previously described as a chromatin boundary element,^4^ and it was shown that it influences nucleosome density and histone gene transcription.^5,6^ The molecular underlying molecular mechanism, however, remained elusive. A idea at the time was that Yta7´s boundary function prevents spreading of repressive chromatin structure into histone gene coding sequences by keeping histone chaperone Rtt106 (Regulator of Ty1 Transposition protein 106) and the chromatin remodeler RSC (Remodeling the Structure of Chromatin) in place.^5^ Another exciting model was that Yta7 actively influences chromatin structure and thus influencing histone gene transcription.^6^ However, there were no biochemical data supporting either hypothesis.

We now showed that Yta7 is a chromatin segregase that uses the energy of ATP hydrolysis to disrupt chromatin structure. Yta7 is very disting from classical chromatin remodelers of the SWI/SNF (SWItch/Sucrose Non-Fermentable) family. It bears two AAA+-ATPase modules, where one is highly conserved among species.^7^ Yta7 assembles into ~1 mega Dalton hexameric complexes that can efficiently bind acetylated chromatin. Interestingly, Yta7 also bears a bromo-domain; however, if this domain is important for the association with acetylated histones is unknown. Interestingly, S-CDK-dependent phosphorylation in close proximity to the AAA+-ATPase domain stimulates ATP hydrolysis and activates the chromatin segegase function of Yta7. As a result, Yta7 disrupts chromatin by disassembling nucleosomes, which facilitates progression of the replisome through a chromatin template, thus preventing replicative stress or genomic instability.^3^

Yta7 has a human homologue ATAD2 (ATPase family AAA+ domain-containing protein 2), which is an emerging oncogene and has been associated with many cancers of different origins.^8,9^ ATAD2 levels in normal proliferating cells are tightly controlled by mitogenic factors via the retinoblastoma (RB) - E2 transcription factor (E2F) pathway. During tumorigenesis, ATAD2 is highly expressed and disturbes the RB-E2F pathway, which contributes to tumor development.^9^ ATAD2 has been reported to function as a transcriptional co-regulator for several oncogenic transcriptional factors such as estrogen receptor or the androgen receptor and the transcription factors E2F and MYC (Master Regulator of Cell Cycle Entry and Proliferative Metabolism).^9^ It has been suggested that ATAD2 might link ocogenic and proliferation pathways and that this drives to the development of aggressive tumors.

Activated oncogenes influence replication by altering replication timing and progression and causes replicative stress but how they accomplish this remains poorly understood.^10^ Interestingly and in line with our findings, ATAD2 has been shown to express in S phase where it then localizes to replication sites. ATAD2 multimerizes through its AAA+-ATPase domain and interacts with acetylated marks on newly synthesis histones to aid heterochromatin compaction. Also, ATAD2 is co-expressed with genes involved in DNA replication in various cancer types,^8^ suggesting that, like Yta7, it might play a role in chromosome replication in multicellular eukaryotes as well.

Because of our discovery and due to the high level of conservation, we propose that ATAD2 might function similarly to Yta7 to influence chromatin structure directly, which is important for faithful and timely chromosome replication.
